# The Chemistry and Therapeutics of Gout
*"On the Chemistry and Therapeutics of Uric Acid Gravel and Gout," being the Croonian Lectures for 1892. With additions. By Sir William Roberts, M.D., F.R.S. Pp. 133. Illustrated. (London : Smith, Elden, and Co., 1892.)


**Published:** 1893-01-14

**Authors:** 


### THE PRACTITIONERS' BOOKSHELF.

### THE CHEMISTRY AND THERAPEUTICS OF GOUT.*

The Croonian Lectures for 1892 can require no introduction to any medical man in the United Kingdom who takes the slightest practical interest in his profession. In the opening lecture Sir William Roberts compares the morbid phenomena associated with uric acid in reference to gravel and calculus and to gout. In the former, uric acid is thrown down in the free state from the urine as concretions in the urinary channels; in the latter, uric acid is thrown down in a state of combination as sodium-biurate in the interior tissues of the body. In regard to gravel and calculus, it is generally conceded that the whole of the morbid sequences are accounted for by the inflammatory incidents and obstructions in the parts implicated; but in regard to gout, the uric acid is supposed to play the double part of causing irritation in the joints and other seats of deposit on the one hand, and also of acting directly as a true poison on the other. But the author shows in the subsequent chapters that it is more probable that uric acid does not possess any truly poisonous quality, and that when mischief arises from it, that mischief is consequent on the acid being precipitated in the solid form as bi-urate in the fluids on tissues of the body.

Of the very interesting amorphous urate deposit the lecturer remarks that we are indebted to Dr. Bence Jones for the first real light on its chemical constitution. He showed that the portion of the sediment which is not dissolved by acting on it with water is pure uric acid, and that the portion which goes into solution is true bi-urate. Bence Jones, as the result of his investigations, concluded that there existed a third order of uric acid salts of more complex constitution than the two previously known, in which an atom of bi-urate was closely combined with an additional atom of uric acid. Sir William Roberts has given these earlier researches a prominent place in his opening lecture, and his views as to the pathology and therapeutics of gout largely turn on the existence of uric acid in the blood in the form of a quadroxalate.

Bi-urates, although known to us pathologically as constituents of gouty concretions, are not certainly known to us as physiological constituents, either of the blood or of the urine. But the quadri-urates exist normally in human urine, and the lecturer demonstrates that when uric acid is brought into relation with the bodily fluids—with the blood, lymph, and synovia—it enters into solution in the first instance, not as neutral urate nor as bi-urate, but as quadri-urate; and thus it appears that whenever and wherever uric acid exists in the healthy body, it exists exclusively as a quadri-urate. The quadri-urate is the physiological or normal form in which uric acid exists in the blood.

In normal acid urine, which is rendered acid by the presence of normal superphosphates, the quadri-urate is first split up into free uric acid and bi-urate. By this reaction half the uric acid is set free. But the bi-urate resulting is immediately transformed in the presence of superphosphate, by a double decomposition, into quadri-urate, which is again split up. Thus, the whole of the uric acid is ultimately set free by the normal decompositions occurring in the urine. If this process takes place too soon, *i.e.*, if it be not retarded till the urine is voided, gravel results. The factors which normally retard this decomposition are reviewed.

In the normal state the uric acid which circulates in the blood as quadri-urate is at once removed unchanged by the kidneys. But in the gouty state, either from defective kidney action or some other cause, the quadri-urate lingers unduly in the blood. The detained quadri-urate is, in a medium which is rich in sodium carbonate, gradually transformed into sodium bi-urate, which is almost insoluble in blood serum, and is, probably for that reason, difficult of removal by the kidneys. Under these new conditions sodium bi-urate accumulates more and more in the blood, and when the accumulation has reached a certain point, is precipitated in the crystalline form in the joints and elsewhere, thereby determining the occurrence of a fit of the gout.

In proof of the accuracy of the view that the quadri-urate undergoes this splitting up in the blood, a standard solution was prepared of the following composition: Sodium chloride, 0·5 grm.; sodium bicarbonate, 0·2 grm.; distilled water, 100 c.c., since for practical purposes it was found that the blood-serum might be regarded as consisting essentially of a solution of these salts in these proportions. Uric acid is freely soluble in this solution, and it enters into solution as a quadri-urate of sodium. The quadri-urate then gradually takes up an additional atom of base, and is thereby converted into bi-urate, and the bi-urate thus formed is, after some delay, eventually precipitated in the crystalline form, for in solutions of sodium salts the bi-urate is very insoluble.

The chapters on the therapeutics and dietetics of gravel and gout are most interesting and very practical, and current views on these important questions are criticised.

* "On the Chemistry and Therapeutics of Uric Acid Gravel and Gout," being the Croonian Lectures for 1892. With additions. By Sir WILLIAM ROBERTS, M.D., F.R.S. Pp. 133. Illustrated. (London: Smith, Elden, and Co., 1892.)

